# Undertreatment of Wernicke encephalopathy in alcohol-related clinical contexts: a scoping review of diagnosis, thiamine regimens, and clinical management

**DOI:** 10.1186/s13722-026-00687-x

**Published:** 2026-10-20

**Authors:** André Akira Sueno Goldani, Gabriel Feitosa, Eduarda Vasconcelos Colchete, Cristhian Ferreira Falleiro, Cristine Weihrauch Pedro, Félix Henrique Paim Kessler, Thiago M. Fidalgo, Lisia von Diemen

**Affiliations:** 1 Centro de Pesquisa em Álcool e Drogas (CPAD), Hospital de Clínicas de Porto Alegre (HCPA), Av. Protásio Alves, 211 – Bloco C – Santa Cecília, Porto Alegre-RS, 90035-903 Brazil; 2 Departamento de Psiquiatria e Medicina Legal, Universidade Federal do Rio Grande do Sul, Porto Alegre, Brazil; 3 Departamento de Psiquiatria, Universidade Federal de São Paulo (UNIFESP), São Paulo, Brazil

**Keywords:** Thiamine, Wernicke encephalopathy, Wernicke–korsakoff syndrome, Alcohol use disorder, Alcohol-related brain damage

## Abstract

**Background:**

Wernicke encephalopathy (WE) is a potentially reversible neurological emergency that remains frequently missed and undertreated in patients with alcohol use disorder (AUD). Although thiamine is widely accepted as the cornerstone of prevention and treatment, diagnostic uncertainty, limited comparative evidence, and heterogeneous clinical recommendations contribute to substantial variability in real-world management. This scoping review aimed to map the available evidence on the diagnosis and clinical management of WE in patients with AUD or clinically significant alcohol use, with particular attention to practice variability, undertreatment, and clinically actionable gaps in care.

**Methods:**

We conducted a scoping review in accordance with PRISMA-ScR guidance. Electronic databases and trial registries were searched for studies addressing the diagnosis, prevention, or treatment of WE in populations with AUD or clinically significant alcohol use. Eligible sources included randomized controlled trials, observational studies, clinical audits, and implementation-focused reports. Data were charted and synthesized thematically.

**Results:**

A total of 26 studies were included. The evidence base was limited and heterogeneous. Only two randomized controlled trials evaluated thiamine regimens, and no consensus emerged regarding optimal dosing. Diagnostic criteria for WE varied widely across studies, with inconsistent use of formal criteria such as the Caine criteria. Across observational studies and prescribing audits, undertreatment was consistently reported, including delayed initiation of therapy and frequent use of oral thiamine in high-risk scenarios. Studies describing more protocolized and guideline-concordant care more often reported earlier treatment initiation and use of parenteral thiamine in suspected or confirmed cases.

**Conclusions:**

WE remains underrecognized and undertreated in AUD and clinically significant alcohol use. The mapped evidence suggests that diagnostic uncertainty, limited trial data, and inconsistent implementation of recommendations are associated with persistent variability in care. Although the optimal thiamine regimen remains unresolved, early recognition and appropriate parenteral treatment in high-risk or suspected cases represent actionable opportunities to reduce preventable neurological morbidity.

**Supplementary Information:**

The online version contains supplementary material available at https://doi.org/10.1186/s13722-026-00687-x.

## Introduction

Alcohol use disorder (AUD) remains a leading cause of preventable morbidity and mortality worldwide, accounting for a substantial proportion of health care expenditure and productivity loss [1]. Chronic ethanol exposure disrupts thiamine homeostasis at multiple levels, including reduced intestinal absorption, depleted hepatic storage, impaired phosphorylation to thiamine pyrophosphate, and frequently inadequate dietary intake [2]. As a result, biochemical or clinical evidence of thiamine deficiency is detected in up to 80% of individuals with AUD [3].

Thiamine is an essential cofactor for key enzymes linking glycolysis to the tricarboxylic acid cycle and the pentose phosphate pathway, including pyruvate dehydrogenase, α-ketoglutarate dehydrogenase, and transketolase. Acute thiamine depletion therefore compromises oxidative ATP production, increases oxidative stress, and predisposes to excitotoxic neuronal injury, particularly in metabolically vulnerable regions such as the thalamus and mammillary bodies [2, 4]. Biochemical assessment of thiamine status has historically relied on markers such as erythrocyte transketolase activity and the thiamine pyrophosphate effect, although these markers are not routinely available in many clinical settings and should be interpreted as supportive evidence rather than stand-alone diagnostic criteria for Wernicke encephalopathy [5].

Clinically, thiamine deficiency may manifest as Wernicke encephalopathy (WE), an acute neuropsychiatric syndrome and potentially reversible neurological emergency. During alcohol withdrawal, the hyperadrenergic state and increased cerebral glucose utilization further elevate thiamine requirements, amplifying the risk and severity of WE when supplementation is delayed or inadequate [2]. In practice, WE is often evaluated in emergency departments, general hospitals, psychiatric units, and medically assisted withdrawal settings, where intoxication, withdrawal, delirium, malnutrition, trauma, infection, and other comorbidities may obscure recognition and delay treatment. When untreated or inadequately treated, WE may progress to Korsakoff syndrome, characterized by severe anterograde amnesia and substantial long-term care needs.

Despite its pathophysiological clarity, WE remains difficult to recognize clinically. The classical triad of encephalopathy, gait ataxia, and ocular motor dysfunction is relatively insensitive: older neuropathological series reported the full triad in only 16–20% of cases, while more recent meta-analytic evidence suggests that it is present in approximately one third of clinically identified cases [6, 7]. Neuropathological studies have also reported WE-related lesions at autopsy in up to 12% of individuals with AUD, including cases not diagnosed during life, highlighting the extent of clinical underrecognition [8]. These autopsy findings should be distinguished from magnetic resonance imaging abnormalities, which have been reported in more than 80% of WE cases in recent meta-analytic evidence [7]. MRI may support the diagnosis, but it is not required for treatment initiation and should not delay empiric thiamine in clinically suspected cases [9].

Expanded diagnostic criteria, such as those proposed by Caine et al., were developed to improve sensitivity beyond the classical triad. These criteria support a diagnosis of WE when at least two of four features are present: dietary deficiency, oculomotor abnormalities, cerebellar dysfunction, and altered mental status or mild memory impairment [10]. Although this broader approach is particularly relevant in patients with AUD, emergency department audits and observational studies continue to document low rates of empiric thiamine administration, even among patients presenting with alcohol-related diagnoses or withdrawal syndromes [11, 12].

At the same time, therapeutic recommendations vary widely. Some guidelines advocate low-dose oral thiamine, whereas others recommend high-dose parenteral regimens for suspected or established WE. This divergence reflects a limited evidence base, with only two randomized controlled trials available, and a reliance on observational data of variable methodological quality [13–15]. Beyond dosing uncertainty, variability also concerns treatment thresholds, the distinction between prophylaxis and treatment, and the choice between oral and parenteral routes.

Previous reviews have addressed thiamine supplementation and WE, but their scopes have differed in terms of populations, included study designs, routes of administration, and attention to prevention, diagnostic criteria, and prescribing practices [16–18]. A synthesis focused specifically on AUD and clinically significant alcohol use is therefore useful for mapping how WE is recognized and managed in practice, where variability arises, and which aspects of care may be most actionable despite persistent uncertainty regarding optimal dosing.

This scoping review aimed to map the available evidence on the diagnosis and clinical management of Wernicke encephalopathy in patients with alcohol use disorder or clinically significant alcohol use, with particular attention to practice variability, undertreatment, and clinically actionable gaps in care.

## Methods

### Protocol registration and reporting standards

The protocol was registered in PROSPERO (CRD42023416281) as a systematic review. The initial review question was designed to assess the evidence on thiamine regimens for the prevention and treatment of WE in populations with AUD or clinically significant alcohol use. During the review process, however, the retrieved literature proved too heterogeneous to support a planned effect-estimation approach, due to variation in study design, diagnostic definitions, clinical settings, thiamine dose, route, duration, and outcome measures. We therefore reframed the work as a scoping review to map the available evidence, characterize practice variability, and identify clinically relevant gaps. The search strategy and eligibility criteria remained unchanged; the main change concerned the synthesis aim and outputs. Reporting follows PRISMA-ScR [19].

### Eligibility criteria

Studies were eligible if they included adults aged 18 years or older diagnosed with alcohol use disorder or classified as having heavy, harmful, or hazardous drinking patterns. To address the recognized underdiagnosis of WE and to enhance generalizability, studies assessing clinically significant alcohol use through screening instruments such as AUDIT or CAGE, or through clinical evaluation, were also included, even when a formal diagnosis of alcohol use disorder was not explicitly reported.

Eligible studies evaluated thiamine interventions for the prevention or treatment of WE, with or without comparators such as placebo, standard care, or alternative regimens. Randomized controlled trials, quasi experimental studies, observational studies including cohort and case control designs, and clinical audits were included, without restrictions on publication date. Non-English studies were translated using machine translation tools, verified by a second reviewer fluent in the language or using back-translation when needed.

Studies involving animals, those reporting outcomes not directly related to the prevention or treatment of WE, and non-generalizable designs such as case reports, narrative reviews, laboratory studies, or conference abstracts were excluded. Studies were also excluded when they lacked sufficient detail on the thiamine intervention to determine its clinical role or regimen, including information on dose, route of administration, frequency, duration, or enough contextual information to classify the intervention as prophylaxis, treatment of suspected or confirmed WE, or maintenance therapy.

### Information sources

The literature search included PubMed, Embase, PsycINFO, Web of Science, CINAHL, the Cochrane Library CENTRAL, Scopus, and ClinicalTrials.gov. Additional searches were conducted in trial registries and reference lists of included studies. The final search was completed on September 19, 2025.

### Search strategy

The search strategy was developed in collaboration with a medical librarian and incorporated controlled vocabulary and free text terms related to thiamine and WE. No restrictions were applied based on language or year of publication. Search strategies were tailored to each database. Reference lists of included studies were manually screened to identify additional relevant articles. Full search strategies are provided in Supplementary Method A.

### Selection process

Study selection was managed using Covidence. Titles and abstracts were independently screened by two pairs of reviewers. Full text articles of potentially eligible studies were assessed independently by the same reviewers, with reasons for exclusion documented at this stage. Calibration exercises were conducted to ensure consistency, and disagreements were resolved through discussion or by involving a third reviewer.

When full text articles were unavailable, multiple attempts were made to contact authors and publishers. Studies that remained inaccessible were documented and excluded, with details provided in Supplementary Method B. For studies with incomplete information regarding thiamine interventions, attempts were made to contact corresponding authors. No additional data were obtained, and such studies were excluded.

### Data collection process

Data were charted using a standardized and calibrated extraction form developed for this review. The form was piloted on five studies and refined iteratively. Two reviewers independently extracted data, with discrepancies resolved by consensus or consultation with a third reviewer. The charting framework was designed to capture population characteristics, diagnostic criteria for alcohol use disorder and WE, details of thiamine interventions, comparators, outcomes, timing, and clinical setting. When biochemical measures of thiamine status were reported as study outcomes or measurement tools, these were extracted within the corresponding outcome and measurement fields.

### Data items

Extracted data items are summarized in Table 1.

**Table 1:** Overview of diagnostic methods and thiamine intervention and outcomes

Category	Data Items/Descriptions
AUD Diagnostic Methods	– Structured Diagnostic Criteria: e.g., DSM, ICD – Screening Tools: e.g., AUDIT or CAGE (without formal criteria) – Inferred Methods: based on patient history, alcohol-related comorbidities, or surrogate markers (e.g., blood alcohol levels) – Not Reported: Method not specified
WE Diagnosis Methods	– Caine Criteria: Requires ≥ 2 of 4 signs: dietary deficiency, ocular abnormalities (e.g., ophthalmoplegia, nystagmus), cerebellar signs (e.g., ataxia), and altered mental status/memory impairment – Classic/ICD Criteria: Based on the classic triad or formal ICD criteria – Inferred Methods: Rely on a single clinical sign, biomarker, or radiological finding (without formal criteria) – Not Reported (NR): Not specified
Intervention Details	– Dosage, administration route (e.g., oral, parenteral), frequency, and duration of treatment
Comparator Details	– Placebo, standard care, or alternative dosing regimens
Outcomes	– Treatment of Confirmed WE: Outcomes related to treated WE cases – Prevention of WE: Outcomes in at-risk populations – Prescribing Practices/Clinical Management: Evaluation of guideline adherence and administration methods – Cognitive, Functional, and Biomarker Outcomes: Effects on cognition, neurological function, and biomarkers
Main Study Findings	– Key conclusions, significant outcomes, implications for clinical practice, noted limitations, and suggestions for future research

### Critical appraisal of included studies

Although critical appraisal is not mandatory for scoping reviews, we assessed risk of bias to describe methodological limitations and contextualize the strength of the evidence, not to determine study eligibility or to rate certainty. We used RoB 2 for randomized trials, ROBINS-I for quasi-experimental studies, and the Newcastle–Ottawa Scale for cohort and cross-sectional studies [20–22].

### Synthesis methods

Given the heterogeneity of study designs, outcomes, and reporting methods, quantitative synthesis was not feasible. Findings were synthesized narratively and organized thematically to map diagnostic approaches, clinical management strategies, prescribing practices, and sources of variability in the recognition and management of WE.

### Reporting bias and certainty assessment

As no quantitative synthesis was performed, formal assessment of reporting bias was not conducted. A formal certainty assessment using tools such as GRADE was not undertaken, as the primary aim of this scoping review was to map diverse evidence and identify gaps rather than to estimate effect sizes. Strengths and limitations of the evidence are discussed narratively.

## Results

### Study selection and study characteristics

**Fig. 1:**
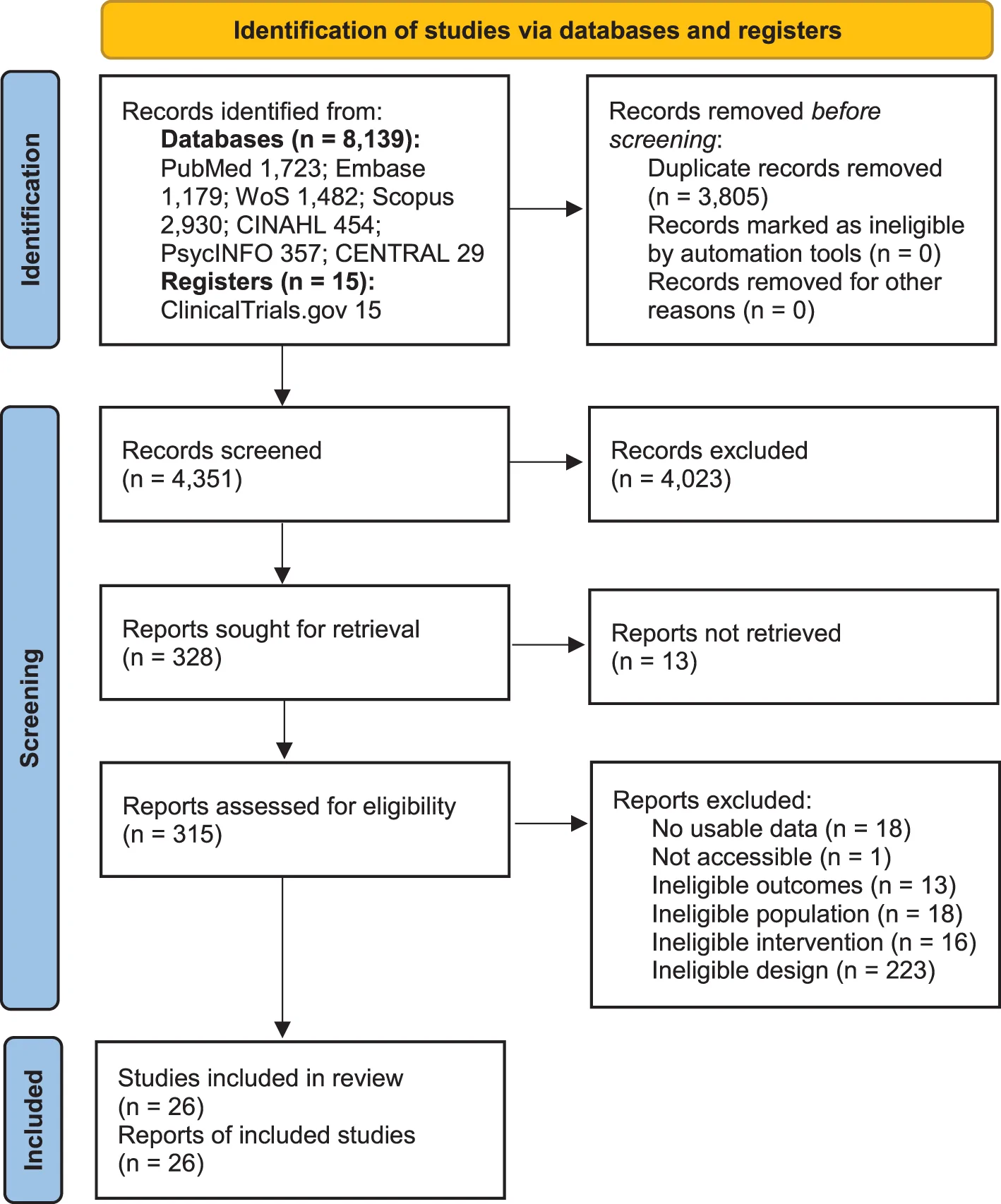
Study selection flow diagram (PRISMA 2020 template), presented for this scoping review in line with PRISMA-ScR reporting guidance. Adapted from Page MJ et al., BMJ 2021;372:n71 (CC BY 4.0) [23]

We included 26 studies comprising 44,240 individuals. After de-duplication, 4,351 records were screened and 4,023 were excluded at title and abstract screening. Of the 328 records advanced to full-text assessment, 13 could not be retrieved despite repeated attempts, leaving 315 full-text articles assessed; 26 met the eligibility criteria (PRISMA flow chart, Fig. 1). The unretrieved articles were all published before 1996, and their titles suggested narrative reviews rather than primary studies (Supplementary Method B and Supplementary Table A). Comprehensive study-level data, including design, diagnostic criteria, thiamine regimens, and outcome measures, are summarized in Table 2, with the full extraction matrix provided in Supplementary Table B.

**Table 2:** Characteristics, findings, and treatment approaches in studies on Wernicke’s encephalopathy and alcohol use disorder

First Author/Year (Country)	Study design	Total n (males%)	AUD Diagnosis	WE Diagnosis	Primary outcomes	Control Conditions	Main Findings or Results*
**Treatment of Confirmed/Suspected Wernicke’s Encephalopathy**							
Damsgaard/2010 (Denmark) [24]	Cross-sectional	497 (61.4%)	Inferred	Inferred	Incidence WE symptoms in alcohol withdrawal patients.	NR	– 11% of admissions had WE – Gait ataxia and cognitive disturbances were most common. – WE patients needed ~50% more phenobarbital.
Baker/1976 (USA) [25]	Quasi-experimental	92 (100%)	Inferred	Inferred	Thiamine activity in blood, RBCs, CSF, and urine with lipid-soluble allithiamines.	Oral allithiamines vs parenteral water-soluble thiamines.	– Lipid-soluble thiamines matched parenteral forms in efficacy and rapidly corrected deficiencies.
Dingwall/2022 (Australia) [26]	RCT	520 (86.1%)	Screening Tools	Caine	Optimum thiamine dosage for preventing/treating WE and WKS.	Low (100 mg/day), intermediate (100 mg thrice daily), and high (300–500 mg thrice daily) dosage groups.	– No major dose-response effect – Minor Story Recall improvement at intermediate doses.
Lin/2020 (USA) [27]	Quasi-experimental	262 (72%)	Inferred	Caine	Prevalence of Caine-positive WKS among psychiatric inpatients; neurocognitive improvement post-IV thiamine.	Untreated Caine-negative psychiatric patients as controls.	– High-dose IV thiamine improved MoCA (+3.6), recall (+1.8), and gait (−0.6) scores vs. untreated group.
Mifsud/2022 (France) [28]	Cohort	56 (82.1%)	Inferred	Caine	Clinical and radiological heterogeneity in thiamine deficiency.	NR	– 54% underwent MRI (63% abnormal) – 59% improved with supplementation. – WE underdiagnosed in non-alcoholics.
Silva/2023 (Portugal) [29]	Cohort	41 (65.9%)	Inferred	Caine	Timing of imaging and thiamine replacement effects on MRI findings in WE.	NR	– High-dose thiamine reduced MRI hyperintensities (defined as ≥200 mg three times a day). – Recovery associated with high dose at admission as well as with a higher total thiamine dose at discharge (*p* = 0.020)
Wood/1977 (Australia) [30]	Cross-sectional	265 (85.3%)	Inferred	Classic/ICD	Biochemical evidence of thiamine deficiency in alcoholics.	NR	– 32.6% had low ETKA – 24.7% elevated TPP – Most achieved full biochemical response despite liver disease.
**Prevention of Wernicke’s Encephalopathy**							
Baron/2024 (USA) [31]	Quasi-experimental	5307 (73.9%)	Formal	NR	Prescribing rates of high-dose thiamine in AUD patients.	Time-based pre-post intervention comparison.	– High-dose thiamine prescriptions rose from 1.1% to 4.7% (AOR 4.50)
Blackmore/2014 (Canada) [32]	Cross-sectional	1000 (70%)	Inferred	Inferred	Delirium prevention via thiamine prophylaxis in trauma patients.	NR	– 17% received thiamine – 44% with elevated BAL and 77% with alcohol abuse received it.
McIntosh/2005 (UK) [33]	Quasi-experimental	163 (71%)	Inferred	Classic/ICD	Adherence to thiamine guidelines post-intervention	Pre- and post-intervention cases to general vs. specialized wards.	– Post-intervention adherence OR: 5.9 – Specialized units OR: 7.0. – Average number of doses increased from 308 mg to 462 mg
Rees/2013 (Australia) [34]	Quasi-experimental	100 (65%)	Formal	Inferred	Mandatory thiamine enrichment impact on blood levels in alcohol-dependent individuals.	Alcohol-dependent individuals (with/without vitamin supplements) compared to controls.	– Non-supplement users had lower thiamine (161 nmol/l vs. 214 nmol/l) – Inpatient thiamine supplementation increased levels significantly, with discharge medians reaching approximately 282–297 nmol/l, thereby normalizing thiamine status
Sinclair/2023 (UK) [35]	Cross-sectional	1255 (65%)	Formal	Inferred	Comparison of mental health vs. addiction units.	Specialist addiction units compared to acute psychiatric wards.	– Specialist units had better adherence to guidelines (88% vs. 41% parenteral thiamine use).
Talbot/2011 (USA) [36]	Quasi-experimental	19 (NR)	Screening Tools	Classic/ICD	Efficacy of oral thiamine in preventing WE in alcohol withdrawal.	NR	– Treatment within 2 hours is optimal. – Oral thiamine (100 mg) corrected delirium – Parenteral needed for poor compliance.
**Prescribing Practices and Clinical Management**							
Novo-Veleiro/2023 (Spain) [37]	Cohort	443 (80.8%)	Inferred	Caine	Thiamine dosage, frequency, and administration effects on WE recovery/mortality.	NR	– 20.9% of patients received an initial thiamine dose > 300 mg/day. – Delays in diagnosis, inadequate treatment regimens, and nutritional deficiencies were associated with worse outcomes, including a 4.9% in-hospital mortality rate
Collet/2019 (France) [38]	Cross-sectional	565 (NR)	Inferred	Inferred	Parenteral thiamine prescribing in alcohol withdrawal patients; effectiveness in complex cases.	NR	– 84.8% reported systematic thiamine prescription during alcohol withdrawal. – When WE symptoms were present, 67.1% used the oral route. – IV administration was favored in internal medicine/surgery/obstetrics services (OR = 18.3, *p* < 0.0001), while psychiatric hospitals preferred IM injections (OR = 4.6, *p* = 0.0353).
Day/2015 (Canada) [39]	Cross-sectional	32213 (NR)	Inferred	Inferred	Parenteral thiamine prescribing rates across university hospitals.	NR	– Parenteral thiamine 57.6% of prescriptions – Protocols increased use (61.3% vs. 45.7%) – ICU favored parenteral.
Ferguson/2000 (UK) [40]	Cross-sectional	218 (97.7%)	Inferred	Inferred	Thiamine administration survey in head injury patients at WKS risk.	NR	– 20.6% at-risk patients received thiamine – Alcohol history influenced use (56.1% alcoholics vs. 26.2% heavy drinkers).
Guirguis/2017 (USA) [41]	Cross-sectional	57 (52.9%)	Inferred	Caine	WE frequency and management in psychiatric inpatients.	NR	– 1.85% had WE; – 7% high-risk. – Most received inadequate doses (oral 100 mg/day)
Isenberg-Grzeda/2014 (USA) [11]	Cross-sectional	217 (71.4%)	Inferred	Inferred	Thiamine prescribing for inpatients with AUD.	NR	– Most received low-dose oral thiamine (50–100 mg). – High-risk patients—defined as those with alcohol intoxication, withdrawal, or delirium tremens—were often left untreated.
Linder/2018 (USA) [42]	Cohort	226 (76%)	Formal	Caine	Thiamine use characterization in AUD patients and WE risk evaluation.	NR	– 89% received oral thiamine – Parenteral thiamine was underutilized to high-risk patients
Nakamura/2018 (USA) [43]	Cohort	432 (70%)	Inferred	Inferred	Thiamine prescribing practices and outcomes in encephalopathy patients.	HDIV (≥200 mg BID) compared to LDIV thiamine.	– HDIV thiamine reduced mortality (2% vs. 13%) – Psychiatric consultation linked to higher dosing.
Ramayya/1997 (UK) [44]	Cohort	47 (59.6%)	Inferred	Classic/ICD	Korsakoff’s psychosis incidence and characteristics in Glasgow (1990–1995).	NR	– WE incidence rose from 12.5/million (1990) to 81.25/million (1995) – Low thiamine response; 85.1% treated, only 4 improved.
**Cognitive, Functional, and Biomarker Studies on Thiamine**							
Ambrose/2001 (Australia) [45]	RCT	107 (NR)	Formal	Inferred	Working memory performance (delayed alternation task)	5, 20, 50, 100, and 200 mg intramuscular thiamine doses in randomised groups.	– 200 mg thiamine dose improved memory more than lower doses – No clear dose-response trend.
Coulbault/2021 (France) [46]	Cross-sectional	50 (60%)	Formal	Inferred	Chronic alcohol consumption effects on thiamine metabolites and cognitive deficits.	Treated and untreated healthy controls.	– AUD patients with severe cognitive impairments had higher thiamine levels – Negative correlation with cognitive scores.
Sgouros/2004 (UK) [47]	Cross-sectional	58 (62.7%)	Formal	Inferred	Development of the Thiamine Deficiency Questionnaire (TDQ).	NR	– TDQ had 73.7% accuracy – Nutritional conditions were key predictors.
Tallaksen/1992 (Norway) [48]	Quasi-experimental	30 (75.4%)	Formal	Classic/ICD	Thiamine/phosphate ester concentrations pre-/post-treatment.	Healthy volunteers receiving the same vitamin injection.	– Alcohol-dependent patients had lower thiamine monophosphate – Levels improved post-supplementation

Legend: AUD (Alcohol Use Disorder), AOR (Adjusted Odds Ratio), BAL (Blood Alcohol Level), ICD (International Classification of Diseases), IV (Intravenous), IM (Intramuscular), MRI (Magnetic Resonance Imaging), MoCA (Montreal Cognitive Assessment), NR (Not Reported), OR (Odds Ratio), RCT (Randomised Controlled Trial), TDQ (Thiamine Deficiency Questionnaire), TX (Treatment), UK (United Kingdom), USA (United States of America), WE (Wernicke’s Encephalopathy), and WKS (Wernicke-Korsakoff Syndrome). *Dosage details are presented in Table 3

Most studies were published in 2000 or later and were conducted exclusively in high-income countries, most commonly in the United States (9 of 26, 34%). Most studies were observational, including 11 cross-sectional and 6 cohort designs, accounting for approximately two thirds of the sample. Seven quasi experimental studies and two randomized controlled trials were identified. Nearly all studies were hospital based, and none enrolled exclusively outpatient populations.

Across these studies, median sample size was 218, ranging from 19 to 32,213 participants. Age was reported in 23 studies and most participants were in their fifth decade of life. Sex distribution was unreported in four studies; among the remaining studies, men accounted for a median of 71% of participants.

Psychiatric comorbidity was reported in ten studies, most commonly mood or anxiety disorders. Medical comorbidities were described in sixteen studies, predominantly neurological conditions such as delirium, traumatic brain injury, and epilepsy.

### Variability in AUD and WE diagnostic approaches

Diagnostic approaches for alcohol use disorder varied substantially. Most studies relied on clinical impression or indirect markers such as alcohol related complications or blood alcohol levels, while a minority applied structured DSM or ICD criteria. Only two intervention studies used validated screening instruments such as AUDIT C or CAGE. Formal grading of AUD severity was reported in fewer than one third of studies.

Case definitions for WE were similarly heterogeneous. Some studies provided no explicit diagnostic criteria. Others applied Caine criteria or classic triad–based definitions or ICD codes. Observational studies more frequently relied on inferred or risk-based diagnoses, whereas interventional studies were evenly divided between inferred and formal diagnostic approaches. This heterogeneity affected both treatment and prevention studies and influenced thresholds for initiating thiamine therapy.

### Mapping thiamine regimens and clinical management strategies

Thiamine regimens varied widely across studies with respect to dose, route of administration, frequency, duration, and study category (Table 3). Because several studies included overlapping aims, these categories reflect the main study focus rather than standardized clinical indications.

**Table 3:** Thiamine treatment regimens: dosage, administration routes, and study characteristics

Study	Design	Population/context	Study category	Dose range (mg/day)	Route(s)	Duration	Key pattern
Ambrose 2001 [45]	RCT	AUD, cognitive outcomes	Cognitive, functional, and biomarker studies	5–200	IM	2 days	Higher IM doses were associated with greater improvement in working memory, although without a clear linear dose–response trend.
Dingwall 2022 [26]	RCT	At-risk vs symptomatic	Prevention and treatment of WE	100–1500	IV	3–5 days	No clear dose–response relationship between low, intermediate and high IV regimens for neurological or cognitive outcomes.
Baker 1976 [25]	Quasi-experimental	Thiamine absorption and biochemical correction in AUD	Treatment of WE/suspected WE	20–50	PO, IV	1–42 days	Lipid-soluble thiamine (TPD) achieved biochemical correction comparable to parenteral thiamine; oral thiamine hydrochloride showed limited effect.
Baron 2024 [31]	Quasi-experimental	Prevention of WE in hospitalized AUD patients	Prevention of WE	100–1500	PO, IV	NR	Protocol-driven care increased high-dose IV thiamine use; low-dose oral maintenance remained frequent.
Lin 2020 [27]	Quasi-experimental	Psychiatric inpatients with suspected WKS	Treatment of WE/suspected WE	200–1200	IV	1–4 days	High-dose IV thiamine was associated with improvements in cognition and gait compared with untreated controls.
McIntosh 2005 [33]	Quasi-experimental	Guideline implementation for WE prevention/treatment	Prevention and treatment of WE	NR	IV	NR	Introduction of prescribing guidance improved adherence to recommended parenteral thiamine use in high-risk patients.
Rees 2013 [34]	Quasi-experimental	Thiamine supplementation and blood levels in AUD	Prevention of WE	100–300	IM, PO	1–10 days	Parenteral thiamine rapidly corrected deficiency; oral thiamine was used for maintenance during inpatient treatment.
Talbot 2011 [36]	Quasi-experimental	Alcohol withdrawal protocol in at-risk patients; “excited delirium” in acute care	Prevention of WE	100	PO, IV	<4 days (IV arm); 30 days (PO arm)	Same nominal daily dose (100 mg) delivered via different routes depending on acuity; oral regimen embedded in longer withdrawal protocol; IV used as rapid inpatient intervention.
Tallaksen 1992 [48]	Quasi-experimental	Biochemical response to thiamine in alcohol dependence	Cognitive, functional, and biomarker studies	105	IM	1 day	Single-dose IM thiamine increased blood thiamine and phosphate ester concentrations.
Blackmore 2014 [32]	Cross-sectional	Trauma patients at risk of delirium	Prevention of WE	100	IV/IM/PO	Mean 5.4 days (1–66)	Thiamine use was inconsistent; route often undocumented, highlighting lack of standardized protocols in acute trauma care.
Collet 2019 [38]	Cross-sectional	National practice survey in AUD	Prescribing practices and clinical management	250–1000	PO, IV, IM	3 days to several weeks	Clear stratification by clinical severity: PO for uncomplicated withdrawal, IV/IM for suspected WE, higher IV doses for complicated cases, followed by long oral maintenance.
Coulbault 2021 [46]	Cross-sectional	AUD patients in addiction treatment entry	Cognitive, functional, and biomarker studies	500	PO	NR	Oral supplementation common; dosing heterogeneous and not clearly linked to clinical severity.
Damsgaard 2010 [24]	Cross-sectional	Alcohol withdrawal patients with/without WE symptoms	Treatment of WE/suspected WE	200–1200	IM, IV	NR	High-dose IV (1200 mg/day) for suspected WE; single IM dose (200 mg) when no neurological signs were present.
Day et al. 2015 [39]	Cross-sectional	Prescribing practices in university hospitals	Prescribing practices and clinical management	100–600	IV, PO	NR	Dominance of low-dose IV (100 mg/day), occasional escalation to high-dose IV (600 mg/day); oral thiamine frequent in psychiatric wards.
Ferguson 2000 [40]	Cross-sectional	Head injury patients at risk of WKS	Prescribing practices and clinical management	25–750	PO, IV	2–5.5 days	Extreme heterogeneity: very low oral doses alongside high IV regimens, suggesting absence of unified guidance.
Guirguis 2017 [41]	Cross-sectional	Psychiatric emergency setting	Prescribing practices and clinical management	100	IV, PO	1 day or NR	Thiamine often given as a single IV dose (“banana bag”) or as oral 100 mg/day, both considered suboptimal for high-risk patients.
Isenberg-Grzeda 2014 [11]	Cross-sectional	General hospital inpatients with AUD	Prescribing practices and clinical management	50–750	PO, IM, IV	NR	Predominance of low-dose oral thiamine; parenteral therapy reserved for a minority despite high-risk presentations.
Sinclair 2023 [35]	Cross-sectional	Acute psychiatric vs specialist addiction units	Prevention of WE	NR	IM, IV, PO	NR	Route strongly depended on availability of specialist care: IM/IV more common with addiction specialists; oral thiamine dominated in general psychiatric wards.
Wood 1977 [30]	Cross-sectional	Biochemical response to thiamine in alcoholism	Treatment of WE/suspected WE	NR	Parenteral	1–9 days (median 1–2)	Focus on metabolic correction rather than dosing protocol; demonstrates early reliance on parenteral replacement.
Linder 2018 [42]	Cohort	Psychiatric hospital patients with AUD, WE risk assessment	Prescribing practices and clinical management	100–1500	PO, IM, IV	NR	Oral 100 mg/day was the default strategy; high-dose IV (500 mg TID = 1500 mg/day) was reserved for suspected WE and was the most common parenteral regimen; IM used as an alternative parenteral route with wide variability (100–600 mg/day).
Mifsud 2022 [28]	Cohort	Tertiary hospital patients with thiamine deficiency/WE	Treatment of WE/suspected WE	<500 to >1000	IV, PO	Variable; PO median 19 days	Clear stepped-care model: initial IV phase with dose escalation (<500 → 500 → 1000 mg/day), followed by oral maintenance. Most patients received high-dose IV (1000 mg/day). Oral maintenance ranged widely, most commonly 501–1000 mg/day.
Nakamura 2018 [43]	Cohort	Hospitalized patients with encephalopathy treated with IV thiamine	Prescribing practices and clinical management	100–1500	IV	NR	Three explicit tiers: low-dose IV (100 mg/day), “high-dose IV” (200 mg BID = 400 mg/day), and guideline-concordant regimen (500 mg TID = 1500 mg/day), the latter used in only 2.1% of cases despite being recommended.
Novo-Veleiro 2023 [37]	Cohort	Multicenter WE cohort evaluating treatment variability and outcomes	Prescribing practices and clinical management	<300 to ≥300	IV, IM, PO	Variable	Dose threshold was prognostic: ≥300 mg/day parenteral associated with better clinical suspicion and outcomes; <300 mg/day linked to delayed diagnosis or milder perceived presentations. Oral 100 mg/day was frequently used but insufficient alone.
Ramayya 1997 [44]	Cohort	Long-term management of Korsakoff psychosis	Prescribing practices and clinical management	125–300	PO, IM, IV	Months to 1 year	Unique chronic-care model: initial parenteral phase (250 mg/day IM/IV for 3–5 days) followed by prolonged maintenance with weekly or biweekly injections, sometimes combined with oral 300 mg/day for years.
Silva 2023 [29]	Cohort	Tertiary hospital WE patients with MRI follow-up	Treatment of WE/suspected WE	600–1500	IV	NR	Two dominant acute strategies: 200 mg TID (600 mg/day) or 500 mg TID (1500 mg/day). High-dose regimens were associated with better MRI resolution and neurological recovery.

Legend AUD, alcohol use disorder; WE, Wernicke encephalopathy; WKS, Wernicke–Korsakoff syndrome; RCT, randomized controlled trial; PO, oral administration; IM, intramuscular; IV, intravenous; QD, once daily; BID, twice daily; TID, three times daily; NR, not reported; TPD, thiamine propyl disulfide; THCI, thiamine hydrochloride; MRI, magnetic resonance imaging; BAL, blood alcohol level; MoCA, Montreal Cognitive Assessment. Study category follows the grouping used in Table 2 and is intended to clarify the context in which thiamine regimens were reported. Some studies included overlapping prevention, treatment, prescribing, or biomarker-related aims; categories therefore reflect the main study focus rather than standardized clinical indications

Reported daily doses ranged from 5 mg to 1,500 mg and were administered orally, intramuscularly, or intravenously. Based on the patterns observed in Table 3, three broad dosing patterns were observed: low-dose regimens of 50 mg per day or less, generally used for prophylaxis or maintenance; intermediate doses of 100 to 200 mg per day across preventive, equivocal, or less clearly defined clinical contexts; and high-dose intravenous regimens of 500 mg per day or more, most often reserved for suspected or clinically established WE.

Once-daily dosing was common, whereas three-times-daily regimens were more often reported in suspected or clinically established WE. Treatment duration mirrored regimen intensity. Short intravenous courses of one to three days were commonly followed by oral maintenance therapy. Parenteral administration predominated in emergency and acute care settings because of superior bioavailability, whereas oral thiamine was more frequently used once patients were clinically stable.

Only a minority of studies explicitly defined what constituted a high-risk clinical scenario, and definitions varied widely, ranging from specific neurological signs to alcohol intake thresholds or Caine criteria. Study design also influenced regimen selection. The two randomized controlled trials followed fixed intravenous protocols of limited duration, whereas observational studies reflected routine clinical practice, combining different routes, doses, and durations according to local protocols and clinician judgment.

### Outcomes assessed across studies

Outcomes were reported across four domains: treatment of confirmed WE, prevention in at-risk populations, prescribing practices and clinical management, and cognitive or biomarker outcomes.

Only two randomized controlled trials were identified. One trial evaluated different intramuscular thiamine doses in AUD patients and reported modest improvements in working memory with higher dosing [45]. The other compared three intravenous thiamine regimens in symptomatic and at risk patients and found no clear dose response differences across cognitive or neurological outcomes [26].

Most treatment-focused studies assessed resolution of classic neurological signs such as ataxia, ocular motor dysfunction, and confusion [24, 27, 29, 30]. A smaller subset incorporated cognitive outcomes [26, 45, 46], biochemical markers of thiamine status [30, 48], or neuroimaging findings [29, 47].

Prevention-oriented studies examined incident WE in high-risk populations such as chronic drinkers, malnourished patients, or individuals undergoing medically assisted withdrawal [32, 33, 35]. Two studies emphasized the importance of early parenteral thiamine administration and identified dietary or clinical risk markers that might prompt prophylactic treatment [32, 34].

Studies evaluating prescribing practices consistently documented low baseline adherence to recommended dosing and routes of administration, particularly limited use of parenteral thiamine in high-risk scenarios [33, 37, 41, 43]. Interventions such as prescribing protocols and electronic order sets were associated with improvements in guideline adherence, although substantial variability persisted across settings [31, 39]. Across study designs and clinical settings, these findings illustrate substantial variability in diagnostic thresholds, thiamine regimens, and prescribing practices.

Biochemical measures of thiamine status were reported in six studies and were used primarily as outcome measures, measurement tools, or supportive indicators rather than stand-alone diagnostic criteria for WE [25, 30, 34, 46–48]. Reported measures included erythrocyte transketolase activity, the thiamine pyrophosphate effect, red blood cell thiamine pyrophosphate, blood or serum thiamine concentrations, thiamine phosphate esters, and urinary thiamine excretion. The inconsistent use and reporting of these measures contributed to heterogeneity in outcome assessment and limited direct comparison across clinical, cognitive, biochemical, and neuroimaging endpoints.

### Methodological limitations of included studies

The two randomized controlled trials were rated at high risk of bias, primarily due to limitations in outcome measurement, attrition, and reporting. Non-randomized studies consistently exhibited substantial risk of bias related to confounding and deviations from intended interventions. Cohort and cross-sectional studies varied in quality, with only a minority achieving high ratings. Detailed assessments are provided in Supplementary Tables C–E and Supplementary Figure A.

## Discussion

This scoping review mapped the available evidence on the diagnosis and clinical management of WE in patients with AUD or clinically significant alcohol use, integrating randomized trials, observational studies, prescribing audits, and implementation-focused reports involving 44,240 individuals. Rather than identifying an optimal thiamine regimen, the findings highlight substantial variability in diagnostic criteria, treatment thresholds, dosing strategies, routes of administration, and implementation of guideline-concordant care. In this review, undertreatment refers primarily to delayed initiation of therapy, use of oral thiamine in high-risk or symptomatic patients, and administration of doses substantially below those recommended in major clinical guidelines and institutional protocols.

Across included studies, thiamine dosing strategies and routes of administration varied markedly, reflecting both the scarcity of robust comparative evidence and the heterogeneity of clinical recommendations. Only two randomized controlled trials were identified, both with important methodological limitations and high risk of bias, limiting their capacity to inform guideline development or resolve dose-response relationships. Observational studies, while providing insight into real-world practice, were similarly constrained by confounding, inconsistent diagnostic definitions, and heterogeneous outcome measurement. These limitations are consistent with prior critiques emphasizing the fragility of the evidence base underpinning current recommendations [18, 49]. Nevertheless, despite unresolved uncertainties regarding optimal dose and duration, routine thiamine supplementation in high-risk populations remains widely supported and biologically plausible [50, 51].

Diagnostic heterogeneity may represent one upstream factor shaping undertreatment, although the available evidence does not allow causal attribution between diagnostic approach and treatment adequacy. Fewer than half of included studies applied formal diagnostic criteria for AUD or WE, with many relying on inferred or risk-based assessments. This heterogeneity mirrors postmortem evidence demonstrating substantial underrecognition of WE in clinical practice [6]. Inconsistent use of expanded criteria, such as those proposed by Caine and colleagues, may further increase the likelihood of missed or delayed recognition, particularly when clinicians rely on the classical triad [10]. These findings suggest that diagnostic ambiguity may contribute to therapeutic inertia, but this interpretation should be understood as hypothesis-generating rather than causal.

Diagnostic heterogeneity also extended to biochemical measures of thiamine status. In the included evidence, biochemical markers were reported in a minority of studies and were used primarily as outcome measures, measurement tools, or supportive indicators rather than stand-alone diagnostic criteria for WE. Reported measures included erythrocyte transketolase activity, the thiamine pyrophosphate effect, red blood cell thiamine pyrophosphate, blood or serum thiamine concentrations, thiamine phosphate esters, and urinary thiamine excretion [25, 30, 34, 46–48]. These markers can support assessment of thiamine status, but their inconsistent use and reporting limited direct comparison across clinical, cognitive, biochemical, and neuroimaging endpoints. This variability also underscores an important distinction: biochemical evidence of thiamine deficiency does not necessarily establish that clinical WE is present.

The relationship between thiamine deficiency and clinical WE is therefore biologically heterogeneous. Thiamine deficiency is central to WE pathophysiology, but available evidence suggests that clinical WE may develop only in a susceptible subgroup of patients with AUD. Camilo, Morgan, and Sherlock reported biochemical thiamine deficiency in patients with alcohol-related liver disease without clinical evidence of WE or peripheral neuropathy, illustrating that biochemical deficiency does not invariably correspond to overt WE [52]. Emerging genetic evidence also suggests that variation in thiamine diphosphate transport may influence susceptibility to Wernicke–Korsakoff syndrome, including variation in SLC19A1 [53]. These findings suggest that thiamine deficiency may interact with individual susceptibility, nutritional status, comorbid illness, and environmental factors to produce the clinical WE phenotype.

Broader nutritional compromise may further contribute to this heterogeneity. Patients with AUD may have deficiencies in other B vitamins and related cofactors, and these may plausibly modify neurological vulnerability in the setting of thiamine deficiency [54, 55]. However, because the present review focused specifically on thiamine interventions and WE-related outcomes, these additional nutritional factors were considered contextual contributors rather than primary targets of the synthesis.

Prescribing practices documented in observational studies and audits reinforce the clinical relevance of this variability. Across settings, low-dose oral thiamine was frequently administered even in acute or high-risk scenarios, despite widespread recommendations favoring parenteral therapy in suspected WE. Such patterns align with concerns raised by Meier and Daeppen, who argued that oral thiamine is insufficient for acute presentations and may compromise outcomes [56]. Importantly, the rarity of serious adverse events associated with parenteral thiamine, including anaphylaxis, supports a low threshold for more assertive treatment approaches in high-risk settings [3, 4]. However, local availability of formulations, institutional protocols, clinician familiarity, and clinical setting continue to shape practice, perpetuating variability across centers [15, 57].

Beyond dosing debates, the mapped literature suggests that practice variability is often shaped by the timeliness of risk recognition and the initiation of parenteral therapy [35, 37, 39]. Across prevention-oriented studies and prescribing audits, earlier parenteral thiamine use in at-risk populations and more timely treatment in suspected cases were commonly reported alongside improved adherence metrics and better documented clinical outcomes in some settings, although causal inference is limited by study design and confounding [27, 31, 33, 37, 43]. From a clinical and implementation perspective, these findings imply that the priority is often not dose refinement alone, but also standardization of diagnostic thresholds, clinician education to support empiric parenteral treatment in high-risk patients, and integration of thiamine protocols into routine workflows, including order sets, admission pathways, and withdrawal protocols.

The evidence base also reveals important gaps in population coverage. Most studies focused on hospitalized, middle-aged men, reflecting the epidemiology of alcohol use disorder but leaving women and older adults underrepresented. These groups may face distinct vulnerabilities related to malnutrition, comorbidities, and barriers to diagnosis, as highlighted in prior work [54, 55, 58]. Addressing these disparities will be essential to ensure equitable prevention and treatment strategies.

Geographic limitations further constrain applicability. All included studies were conducted in high-income countries, despite the disproportionate burden of nutritional deficiencies, delayed diagnosis, and limited access to parenteral thiamine or specialized diagnostic resources in low- and middle-income settings. Public health approaches such as food fortification and targeted supplementation programs, successfully implemented in countries such as Australia, illustrate the potential of population-level strategies to reduce WE [50]. Expanding such initiatives requires coordinated global efforts and adaptation to resource-constrained environments [55]. The absence of studies from low- and middle-income countries represents a critical blind spot.

Importantly, uncertainty regarding individual susceptibility should not reduce the clinical priority of thiamine replacement. Because clinicians cannot reliably identify which thiamine-deficient or high-risk patients will progress to WE, and because parenteral thiamine is generally safe, the mapped evidence supports early recognition and a low threshold for parenteral treatment in high-risk or clinically suspected cases. The most actionable gaps identified by this review therefore concern not only the optimal dose, but also earlier recognition, clearer treatment thresholds, and more consistent implementation of guideline-concordant thiamine protocols across acute-care, psychiatric, and addiction-treatment settings.

### Limitations

This scoping review has several limitations that should be interpreted in light of both the nature of the available evidence and the objectives of a scoping approach. Many included studies were retrospective, single-center investigations with small sample sizes and heterogeneous outcome measures, reflecting the limited methodological maturity of this field. Variability in diagnostic definitions, dosing regimens, and treatment duration limited direct comparability across studies but also represents a central finding of this review, namely the absence of standardized diagnostic and therapeutic frameworks for WE. Long-term outcomes, including cognitive recovery and recurrence of WE, were infrequently assessed, highlighting an important gap in the literature rather than a shortcoming of this synthesis alone. Although risk of bias was systematically described, the predominance of observational evidence restricts causal inference, which is inherent to the current evidence base. Finally, despite an extensive and librarian-assisted search strategy, 13 pre-1996 articles could not be retrieved; these were mostly non-English publications and, based on their titles, appeared to be narrative reviews rather than primary studies. Their exclusion is therefore unlikely to have materially affected the mapping of original evidence but illustrates the challenges posed by older and inconsistently indexed literature.

### Future directions

Future research should prioritize the development and adoption of standardized diagnostic frameworks for WE in patients with AUD or clinically significant alcohol use, integrating clinical criteria with supportive markers when available, such as biochemical measures of thiamine status and characteristic neuroimaging findings [3, 10]. Future studies should clearly distinguish thiamine deficiency, suspected WE, confirmed WE, and Korsakoff syndrome when defining populations and outcomes, because these constructs do not fully overlap clinically, biochemically, or radiologically.

Well-powered pragmatic trials are needed to evaluate treatment strategies across clinically relevant scenarios, focusing not only on dose comparisons but also on timing, route of administration, duration, and implementation within routine care pathways [18]. Emerging evidence suggesting that thiamine supplementation may confer additional benefits in AUD populations, including cognitive improvement and reduced infection-related mortality, warrants further investigation [31, 59, 60].

At the public health level, renewed evaluation of fortification and targeted supplementation strategies is needed, particularly in settings with high prevalence of malnutrition and limited access to parenteral therapy [49, 54]. Greater international coordination is necessary to harmonize case definitions, treatment protocols, and surveillance efforts, especially in low- and middle-income countries and other resource-constrained settings [55, 57, 61].

## Conclusions

This scoping review shows that WE remains underrecognized and undertreated in individuals with AUD or clinically significant alcohol use, despite being a preventable and potentially reversible neurological emergency. The evidence base is marked by substantial heterogeneity in diagnostic criteria, thiamine regimens, clinical settings, outcome measures, and prescribing practices, alongside limited randomized evidence.

The mapped evidence suggests that undertreatment is associated with diagnostic ambiguity, delayed recognition of risk, reliance on oral thiamine in high-risk or symptomatic patients, and inconsistent implementation of existing recommendations. Although the optimal thiamine regimen remains unresolved, early recognition and appropriate parenteral treatment in high-risk or suspected cases emerge as actionable priorities.

Improving care for WE is likely to require not only better dose-comparison evidence, but also clearer diagnostic thresholds, more consistent use of guideline-concordant protocols, and stronger implementation in acute-care, psychiatric, and addiction-treatment settings. These steps represent immediate opportunities to reduce preventable neurological morbidity while more definitive comparative evidence is generated.

## Electronic supplementary material

Below is the link to the electronic supplementary material.

Supplementary Material 1

## Data Availability

The dataset(s) supporting the conclusions of this article are included within the article and its supplementary information files.

## Declarations

### Generative AI and AI-assisted technologies in the manuscript preparation process

During the preparation of this manuscript, the authors used ChatGPT (OpenAI) exclusively for language editing and style improvement. The tool was not used to generate or modify scientific content, interpret data, perform analyses, determine eligibility, extract data, or make methodological decisions. The authors reviewed and edited all text and take full responsibility for the accuracy and integrity of the work.
