# Supplementary material for: Undertreatment of Wernicke encephalopathy in alcohol-related clinical contexts: a scoping review of diagnosis, thiamine regimens, and clinical management
Source: Addict Sci Clin Pract. 2026 Oct 20;21:80. doi: 10.1186/s13722-026-00687-x (PMC13681891; doi:10.1186/s13722-026-00687-x)
Supplement: Supplementary file 1 [file 13722_2026_687_MOESM1_ESM.docx]

**Supplementary Material**

**Undertreatment of Wernicke encephalopathy in alcohol-related clinical contexts: a scoping review of diagnosis, thiamine regimens, and clinical management**

**Supplementary Method A. Database Search Strategies (through September 19, 2025)**

- Full reproducible strategies for PubMed/MEDLINE, Embase, PsycInfo, Web of Science, CINAHL, Scopus, CENTRAL, and ClinicalTrials.gov. No language or date restrictions.

**PubMed**

(Thiamine[mh] OR Thiamin*[tiab] OR Vitamin B1[tiab] OR Aneurin*[tiab] OR Vitamin B 1[tiab] OR Vitamin B-1[tiab] OR Fursultiamin*[tiab] OR Cocarboxylas*[tiab] OR Berolas*[tiab] OR Tiamin*[tiab] OR Thyamin*[tiab] OR benerva[tiab] OR beneuran[tiab] OR beneurin[tiab] OR betabion[tiab] OR betalin[tiab] OR betaxin[tiab] OR bethiamin[tiab] OR bevitine[tiab] OR bewon[tiab] OR biamine[tiab] OR "eskay's theranates"[tiab] OR metabolin[tiab] OR pabiamine[tiab] OR sheramin[tiab] OR vibex[tiab])

AND

(Wernicke Encephalopathy[mh] OR Wernick*[tiab] OR Cerebral Beriberi*[tiab] OR Gayet-Wernick*[tiab] OR Korsakoff Syndrome[mh] OR Korsakoff*[tiab] OR "acute haemorrhagic polioencephalitis"[tiab] OR "acute hemorrhagic polioencephalitis"[tiab] OR "encephalopathia haemorrhagica"[tiab] OR "encephalopathia hemorrhagica"[tiab] OR "encephalopathy haemorrhagic superior"[tiab] OR "encephalopathy hemorrhagic superior"[tiab] OR "polioencephalopathia haemorrhagica superior"[tiab] OR "polioencephalopathia hemorrhagica superior"[tiab] OR "polioencephalopathy haemorrhagic superior"[tiab] OR "polioencephalopathy hemorrhagic superior"[tiab])

**Embase**

('thiamine'/exp OR (Thiamin* OR 'Vitamin B1' OR Aneurin* OR 'Vitamin B 1' OR 'Vitamin B-1' OR Fursultiamin* OR Cocarboxylas* OR Berolas* OR Tiamin* OR Thyamin* OR benerva OR beneuran OR beneurin OR betabion OR betalin OR betaxin OR bethiamin OR bevitine OR bewon OR biamine OR 'eskay`s theranates' OR metabolin OR pabiamine OR sheramin OR vibex):ti,ab,kw)

AND

('Wernicke encephalopathy'/exp OR 'Wernicke Korsakoff syndrome'/exp OR 'Korsakoff psychosis'/exp OR (Wernick* OR 'Cerebral Beriberi*' OR 'Gayet-Wernick*' OR Korsakoff* OR 'acute haemorrhagic polioencephalitis' OR 'acute hemorrhagic polioencephalitis' OR 'encephalopathia haemorrhagica' OR

'encephalopathia hemorrhagica' OR 'encephalopathy haemorrhagic superior' OR 'encephalopathy hemorrhagic superior' OR 'polioencephalopathia haemorrhagica superior' OR 'polioencephalopathia hemorrhagica superior' OR 'polioencephalopathy haemorrhagic superior' OR 'polioencephalopathy hemorrhagic superior'):ti,ab,kw)

AND

[embase]/lim NOT ([embase]/lim AND [medline]/lim)

**PsycInfo**

((IndexTermsFilt: ("Wernicke Encephalopathy") OR IndexTermsFilt: ("Korsakoff Syndrome")) OR (title: (Wernick*) OR title: ("Cerebral Beriberi*") OR title:

("Gayet-Wernick*") OR title: (Korsakoff*) OR title: ("acute haemorrhagic polioencephalitis") OR title: ("acute hemorrhagic polioencephalitis") OR title:

("encephalopathia haemorrhagica") OR title: ("encephalopathia hemorrhagica") OR title: ("encephalopathy haemorrhagic superior") OR title: ("encephalopathy hemorrhagic superior") OR title: ("polioencephalopathia haemorrhagica superior") OR title: ("polioencephalopathia hemorrhagica superior") OR title: ("polioencephalopathy haemorrhagic superior") OR title: ("polioencephalopathy hemorrhagic superior")) OR (abstract: (Wernick*) OR abstract: ("Cerebral Beriberi*") OR abstract: ("Gayet-Wernick*") OR abstract: (Korsakoff*) OR abstract: ("acute haemorrhagic polioencephalitis") OR abstract: ("acute hemorrhagic polioencephalitis") OR abstract: ("encephalopathia haemorrhagica") OR abstract: ("encephalopathia hemorrhagica") OR abstract: ("encephalopathy haemorrhagic superior") OR abstract: ("encephalopathy hemorrhagic superior") OR abstract: ("polioencephalopathia haemorrhagica superior") OR abstract: ("polioencephalopathia hemorrhagica superior") OR abstract: ("polioencephalopathy haemorrhagic superior") OR abstract: ("polioencephalopathy hemorrhagic superior")) OR (Keywords: (Wernick*) OR Keywords: ("Cerebral Beriberi*") OR Keywords: ("Gayet-Wernick*") OR Keywords: (Korsakoff*) OR Keywords: ("acute haemorrhagic polioencephalitis") OR Keywords: ("acute hemorrhagic polioencephalitis") OR Keywords: ("encephalopathia haemorrhagica") OR Keywords: ("encephalopathia hemorrhagica") OR Keywords: ("encephalopathy haemorrhagic superior") OR Keywords: ("encephalopathy hemorrhagic superior") OR Keywords: ("polioencephalopathia haemorrhagica superior") OR Keywords: ("polioencephalopathia hemorrhagica superior") OR Keywords: ("polioencephalopathy haemorrhagic superior") OR Keywords: ("polioencephalopathy hemorrhagic superior")))

AND

((title: (Thiamin*) OR title: ("Vitamin B1") OR title: (Aneurin*) OR title: ("Vitamin B 1") OR title: ("Vitamin B- 1") OR title: (Fursultiamin*) OR title: (Cocarboxylas*) OR title: (Berolas*) OR title: (Tiamin*) OR title: (Thyamin*) OR title: (benerva) OR title: (beneuran) OR title: (beneurin) OR title: (betabion) OR title: (betalin) OR title: (betaxin) OR title: (bethiamin) OR title: (bevitine) OR title: (bewon) OR title: (biamine) OR title: ("eskay`s theranates") OR title: (metabolin) OR title: (pabiamine) OR title: (sheramin) OR title: (vibex)) OR (abstract: (Thiamin*) OR abstract: ("Vitamin B1") OR abstract: (Aneurin*) OR abstract: ("Vitamin B 1") OR abstract: ("Vitamin B-1") OR abstract: (Fursultiamin*) OR abstract: (Cocarboxylas*) OR abstract: (Berolas*) OR abstract: (Tiamin*) OR abstract: (Thyamin*) OR abstract: (benerva) OR abstract: (beneuran) OR abstract: (beneurin) OR abstract: (betabion) OR abstract:

(betalin) OR abstract: (betaxin) OR abstract: (bethiamin) OR abstract: (bevitine) OR abstract: (bewon) OR abstract: (biamine) OR abstract: ("eskay`s theranates") OR abstract: (metabolin) OR abstract: (pabiamine) OR abstract: (sheramin) OR abstract: (vibex)) OR (Keywords: (Thiamin*) OR Keywords: ("Vitamin B1") OR Keywords: (Aneurin*) OR Keywords: ("Vitamin B 1") OR Keywords: ("Vitamin B-1") OR Keywords: (Fursultiamin*) OR Keywords: (Cocarboxylas*) OR Keywords: (Berolas*) OR Keywords: (Tiamin*) OR Keywords: (Thyamin*) OR Keywords: (benerva) OR Keywords: (beneuran) OR Keywords: (beneurin) OR Keywords: (betabion) OR Keywords: (betalin) OR Keywords: (betaxin) OR Keywords: (bethiamin) OR Keywords: (bevitine) OR Keywords: (bewon) OR Keywords: (biamine) OR Keywords: ("eskay`s theranates") OR Keywords: (metabolin) OR Keywords: (pabiamine) OR Keywords: (sheramin) OR Keywords: (vibex)))

**Web of Science**

TS=(Thiamin* OR "Vitamin B1" OR Aneurin* OR "Vitamin B 1" OR "Vitamin B-1" OR Fursultiamin* OR Cocarboxylas* OR Berolas* OR Tiamin* OR Thyamin* OR benerva OR beneuran OR beneurin OR betabion OR betalin OR betaxin OR bethiamin OR bevitine OR bewon OR biamine OR "eskay`s theranates" OR metabolin OR pabiamine OR sheramin OR vibex)

AND

TS=(Wernick* OR "Cerebral Beriberi*" OR "Gayet-Wernick*" OR Korsakoff* OR "acute haemorrhagic polioencephalitis" OR "acute hemorrhagic polioencephalitis" OR "encephalopathia haemorrhagica" OR "encephalopathia hemorrhagica" OR "encephalopathy haemorrhagic superior" OR "encephalopathy hemorrhagic superior" OR "polioencephalopathia haemorrhagica superior" OR "polioencephalopathia hemorrhagica superior" OR "polioencephalopathy haemorrhagic superior" OR "polioencephalopathy hemorrhagic superior")

**CINAHL**

(MH Thiamine OR TI ( Thiamin* OR "Vitamin B1" OR Aneurin* OR "Vitamin B 1" OR "Vitamin B-1" OR Fursultiamin* OR Cocarboxylas* OR Berolas* OR Tiamin* OR Thyamin* OR benerva OR beneuran OR beneurin OR betabion OR betalin OR betaxin OR bethiamin OR bevitine OR bewon OR biamine OR "eskay`s theranates" OR metabolin OR pabiamine OR sheramin OR vibex ) OR AB ( Thiamin* OR "Vitamin B1" OR Aneurin* OR "Vitamin B 1" OR "Vitamin B-1" OR Fursultiamin* OR Cocarboxylas* OR Berolas* OR Tiamin* OR Thyamin* OR benerva OR beneuran OR beneurin OR betabion OR betalin OR betaxin OR bethiamin OR bevitine OR bewon OR biamine OR "eskay`s theranates" OR metabolin OR pabiamine OR sheramin OR vibex ) OR SU ( Thiamin* OR "Vitamin B1" OR Aneurin* OR "Vitamin B 1" OR "Vitamin B-1" OR Fursultiamin* OR Cocarboxylas* OR Berolas* OR Tiamin* OR Thyamin* OR benerva OR beneuran OR beneurin OR betabion OR betalin OR betaxin OR bethiamin OR bevitine OR bewon OR biamine OR "eskay`s theranates" OR metabolin OR pabiamine OR sheramin OR vibex ))

AND

(MH ( "Wernicke Encephalopathy" OR "Korsakoff Syndrome" ) OR TI ( Wernick* OR "Cerebral Beriberi*" OR "Gayet-Wernick*" OR Korsakoff* OR "acute haemorrhagic polioencephalitis" OR "acute hemorrhagic polioencephalitis" OR "encephalopathia haemorrhagica" OR "encephalopathia hemorrhagica" OR "encephalopathy haemorrhagic superior" OR "encephalopathy hemorrhagic superior" OR "polioencephalopathia haemorrhagica superior" OR "polioencephalopathia hemorrhagica superior" OR "polioencephalopathy haemorrhagic superior" OR "polioencephalopathy hemorrhagic superior" ) OR AB ( Wernick* OR "Cerebral Beriberi*" OR "Gayet-Wernick*" OR Korsakoff* OR "acute haemorrhagic polioencephalitis" OR "acute hemorrhagic polioencephalitis" OR "encephalopathia haemorrhagica" OR "encephalopathia hemorrhagica" OR "encephalopathy haemorrhagic superior" OR "encephalopathy hemorrhagic superior" OR "polioencephalopathia haemorrhagica superior" OR "polioencephalopathia hemorrhagica superior" OR "polioencephalopathy haemorrhagic superior" OR "polioencephalopathy hemorrhagic superior" ) OR SU ( Wernick* OR "Cerebral Beriberi*" OR "Gayet-Wernick*" OR Korsakoff* OR "acute haemorrhagic polioencephalitis" OR "acute hemorrhagic polioencephalitis" OR "encephalopathia haemorrhagica" OR "encephalopathia hemorrhagica" OR "encephalopathy haemorrhagic superior" OR "encephalopathy hemorrhagic superior" OR "polioencephalopathia haemorrhagica superior" OR "polioencephalopathia hemorrhagica superior" OR "polioencephalopathy haemorrhagic superior" OR "polioencephalopathy hemorrhagic superior" ))

**Scopus**

TITLE-ABS-KEY(Thiamin* OR "Vitamin B1" OR Aneurin* OR "Vitamin B 1" OR "Vitamin B-1" OR Fursultiamin* OR Cocarboxylas* OR Berolas* OR Tiamin* OR

Thyamin* OR benerva OR beneuran OR beneurin OR betabion OR betalin OR betaxin OR bethiamin OR bevitine OR bewon OR biamine OR "eskay`s theranates" OR metabolin OR pabiamine OR sheramin OR vibex)

AND

TITLE-ABS-KEY(Wernick* OR "Cerebral Beriberi*" OR "Gayet-Wernick*" OR Korsakoff* OR "acute haemorrhagic polioencephalitis" OR "acute hemorrhagic

polioencephalitis" OR "encephalopathia haemorrhagica" OR "encephalopathia hemorrhagica" OR "encephalopathy haemorrhagic superior" OR "encephalopathy hemorrhagic superior" OR "polioencephalopathia haemorrhagica superior" OR "polioencephalopathia hemorrhagica superior" OR "polioencephalopathy haemorrhagic superior" OR "polioencephalopathy hemorrhagic superior")

**CENTRAL**

ID Search Hits

#1 MeSH descriptor: [Thiamine] explode all trees 344

#2 Thiamin* OR "Vitamin B1" OR Aneurin* OR "Vitamin B 1" OR "Vitamin B-1" OR Fursultiamin* OR Cocarboxylas* OR Berolas* OR Tiamin* OR Thyamin* OR benerva OR beneuran OR beneurin OR betabion OR betalin OR betaxin OR bethiamin OR bevitine OR bewon OR biamine OR "eskay`s theranates" OR metabolin OR pabiamine OR sheramin OR vibex 1296

#3 #1 OR #2 1296

#4 MeSH descriptor: [Wernicke Encephalopathy] explode all trees 6

#5 MeSH descriptor: [Korsakoff Syndrome] explode all trees 13

#6 Wernick* OR "Cerebral Beriberi*" OR "Gayet-Wernick*" OR Korsakoff* OR "acute haemorrhagic polioencephalitis" OR "acute hemorrhagic polioencephalitis" OR "encephalopathia haemorrhagica" OR "encephalopathia hemorrhagica" OR "encephalopathy haemorrhagic superior" OR "encephalopathy hemorrhagic superior" OR "polioencephalopathia haemorrhagica superior" OR "polioencephalopathia hemorrhagica superior" OR "polioencephalopathy haemorrhagic superior" OR "polioencephalopathy hemorrhagic superior" 362

#7 #4 OR #5 OR #6 362

#8 #3 AND #7 26

**Supplementary Method B. Retrieval of Unavailable Full Texts**

We attempted to retrieve 13 legacy/non-digitized records via a standardized process: (i) checked publisher sites and library holdings (no digitized PDFs), (ii) searched authors on ResearchGate/Google Scholar and emailed available contacts, and (iii) requested copies from journal offices. Outcome: 0/13 obtained. Most records were non-English and pre-2000; several appeared to be reviews (an exclusion criterion). Full details appear in **Supplementary Table A**.

**Supplementary Table A: Characteristics of Articles Not Retrieved Despite Retrieval Efforts**

| **Publication Year** | **Author** | **Title** | **Publication Title** | **Pages** | **Issue** | **Volume** | **Language** |
| --- | --- | --- | --- | --- | --- | --- | --- |
| 1964 | VILLARD, H.P.; BEAUREGARD, J.M. | DEFICIENCY ALCOHOLIC ENCEPHALOPATHY AND MAGNESIUM: GAYET-WERNICKE SYNDROME. | L'unión médicale du Canada | 50-53 | - | 93 | French |
| 1969 | Silfverskiöld, B P | The Wernicke-Korsakoff syndrome and ataxia in alcoholics | Lakartidningen | 4773-4778 | 46 | 66 | Swedish |
| 1977 | Itokawa, Y. | Thiamine (Vitamin B1) and diseases related to vitamin B1 deficiencies | Journal of Applied Nutrition | 5-16 | 1-2 | 29 | English |
| 1977 | Neundörfer, B; Gössinger, S | Clinical diagnosis and course of wernicke's encephalopathy (author's transl) | Nervenarzt | 500-504 | 9 | 48 | German |
| 1978 | Laplane, D. | Indications for vitamin B therapy in neurology | Revue de Medecine de Tours | 657-659 | 5 | 12 | French |
| 1978 | Silfverskiold, B. | Vitamin B1 deficient enzyme anomaly in Wernicke Korsakoff patients | Lakartidningen | 294-295 | 5 | 75 | Swedish |
| 1980 | Waldenlind, L. | Thiamine and thiamine deficiency in alcoholics | Lakartidningen | 1536-1538 | 16 | 77 | Swedish |
| 1981 | Woerz, R. | Alcohol withdrawal syndrome and immediate treatment | Notfall Medizin | 545-548 | 5 | 7 | German |
| 1983 | Fontanari, D.; Paladin, F.; Russo Perez, G.; Fassetta, G.; Tambato, E.; Toso, V.; Zanetti, R.; Cocco, G.; Colles, L. | Nutritional neurologic pathology in the alcoholic | Rivista di Neurobiologia | 208-228 | 2-3 | 29 | Italian |
| 1988 | Iwata, M. | Central nervous system diseases associated with alcoholism | Nippon rinsho. Japanese journal of clinical medicine | 1686-1690 | 8 | 46 | Japanese |
| 1990 | Jouanny, P.; Barroche, G. | Nutritional neurological diseases. 1. Nutritional diseases related to alcoholism | Gazette Medicale | 31-34 | 38 | 97 | French |
| 1991 | Lindenstrøm, E S; Christiansen, L W; Simonsen, E | [Wernicke-Korsakoff syndrome at the Rikshospitalet in 1979-1988. A retrospective study] | Ugeskr Laeger | 2819-2822 | 40 | 153 | Danish |
| 1996 | Moltó, J M | [Neurological complications associated with vitamin B1 deficiency(thiamine)] | Rev Neurol | 389-391 | 128 | 24 | Spanish |

**Supplementary Table B. Characteristics and Outcome Data of Included Studies**

| **First Author**  **Publication year** | **Age (mean ± SD)** | **Clinical context (Inpatient, Outpatient)** | **Specified Psychiatric comorbidities** | **Specified Non-Psychiatric Comorbidities** | **Alcohol Diagnosis Method Details** | **Alcohol Severity Quantification Method** | **AUD severity according to authors** | **Wernicke encephalopathy diagnosis criteria details** | **Primary Outcomes** | **Secondary Outcomes** | **Tools and Measurements** | **Observations and Limitations** |
| --- | --- | --- | --- | --- | --- | --- | --- | --- | --- | --- | --- | --- |
| **Ambrose**  2001 | 42 | Inpatient | NR | NR | DSM-IV diagnostic criteria | Daily alcohol intake + duration of alcohol use | Severe | Inferred Methods (ataxia, eye movement abnormalities, and cognitive performance) | Thiamine dose effects on working memory in alcohol-dependent individuals. | Dosage effects on delayed alternation tasks. | MMSE, delayed alternation task. | High noncompletion rate; small groups; short treatment duration; no clear dose-response relationship. |
| **Baker**  1976 | NR | Inpatient | NR | Liver disease, neurological abnormalities, hypovitaminosis | Hypovitaminosis, presence of liver and neurological diseases, biomarkers (RBC transketolase activity) | NA | NR | Inferred Methods (clinical signs: e.g., lateral rectos palsy, confabulation, ataxia, and peripheral neuropathy) | Thiamine activity in blood, RBCs, CSF, and urine with lipid-soluble allithiamines. | Restoration of transketolase activity in alcoholics. | Thiamine activity assays, RBC transketolase, urine analysis, Ochromonas danica bioassay. | Single-center study; predominantly male sample; absorption differences between water- and lipid-soluble thiamines. |
| **Baron**  2024 | 55.8 ± 14.1 | Inpatient | NR | NR | ICD-9 codes to identify alcohol-related diagnoses | NA | NR | Inferred Methods (it infers the risk of WE based on patient populations defined by alcohol-related diagnoses (using ICD-9 codes) and elevated alcohol levels (≥80 mg/dL) | Prescribing rates of high-dose thiamine in AUD patients. | Time-to-prescription of high-dose thiamine. | EHR-based intervention, Statistical Process Control Charts, Kaplan-Meier plots. | Single-center design; lack of educational interventions; non-randomized; limited optimal prescribing data. |
| **Blackmore**  2014 | 48,18 ± 21,59 | Inpatient | Suicide attempt (2,5% of sample) | Severe trauma injury | Blood alcohol level (BAL) testing at admission, with specific thresholds to identify intoxication and assess chronic alcohol use potential. Documentation of a history of alcohol abuse in patient medical records. | NA | NR | Inferred Methods (WE risk, focusing on clinical signs, blood alcohol level (BAL) monitoring, and the administration of thiamine as a preventive measure) | Delirium prevention via thiamine prophylaxis in trauma patients. | WE incidence in alcohol abuse and malnutrition. | Blood alcohol level (BAL), Alberta Trauma Registry, electronic medical records. | Small subgroup sizes; no standard protocols; electronic documentation issues. |
| **Collet**  2019 | NR | Both | NR | NR | General patient history of alcohol consumption, complications like liver disease, or observed cognitive deficits | NA | NR | Inferred Methods (suspected WE based on signs such as malnutrition, cognitive impairment, or alcohol-induced health conditions) | Parenteral thiamine prescribing in alcohol withdrawal patients; effectiveness in complex cases. | Prescription factors (e.g., care center type, practitioner experience). | Self-administered questionnaire, Caine criteria for WE, statistical tools. | Regional sample; practice/training variability; self-reported data; no standardized criteria across centers. |
| **Coulbault**  2019 | 45.2 ± 9.4 (of the AUD group) | Both | Cognitive impairment | NR | DSM-IV | AUDIT score | Severe | Inferred Methods (The Brief Evaluation of Alcohol-Related Neuropsychological Impairments (BEARNI) and the  Montreal Cognitive Assessment (MoCA) scores) | Chronic alcohol consumption effects on thiamine metabolites and cognitive deficits. | Thiamine metabolism and neuropsychological relationships. | BEARNI, MoCA, liquid chromatography with fluorimetric detection. | Small sample; no baseline thiamine levels; dietary variations; unreported prior supplementation. |
| **Damsgaard**  2010 | 52.0 ± 7.1 | Inpatient | NR | NR | Assessment of alcohol consumption history and withdrawal symptoms during hospital admissions for detoxification | NA | NR | Inferred Methods (presence of at least one of the classic triad symptoms) | Incidence of Wernicke's Encephalopathy (WE) symptoms in alcohol withdrawal patients. | Drinking history prior to admission; impact of phenobarbital on WE incidence. | Clinical examination, phenobarbital dosage, retrospective alcohol consumption history. | Descriptive study; clinical diagnosis prone to over-reporting; small subset with retrospective drinking data. |
| **Day**  2015 | NR | Inpatient | NR | NR | Patients at risk of thiamine deficiency; alcohol use not explicitly stated but inferable as a primary cause due to study context | NA | NR | Inferred Methods (While the study mentions populations at risk of thiamine deficiency (e.g., alcohol misuse, malnourished patients), it does not describe a formal diagnostic approach or criteria used to confirm WE in these patients.) | Parenteral thiamine prescribing rates across university hospitals. | Protocol impact on prescribing patterns. | Pharmacy information systems. | Limited to Canadian academic hospitals; anonymized data; non-generalizable to non-academic settings. |
| **Dingwall**  2022 | 43 ± 11,55 | Inpatient | NR | NR | AUDIT-C > 4 | Daily alcohol intake + AUDIT-C score | Mild | Caine Criteria | Optimum thiamine dosage for preventing/treating WE and WKS. | Neurological function improvements (ataxia, oculomotor issues, confusion). | RUDAS, CogState tests, Story Recall Memory Test, neurological assessments. | High attrition; small symptomatic group; baseline variability; treatment adherence ranged from 39–93%. |
| **Ferguson**  2000 | 42,2 | Inpatient | NR | Traumatic brain injury (100%) | Alcohol consumption history recorded in patient notes, including history of drinking prior to injury, breath smelling of alcohol, positive alcometer reading, or positive blood-alcohol level. | NA | NR | Inferred Methods (clinical signs and associated risk factors (e.g., administration of intravenous glucose without thiamine supplementation) | Thiamine administration survey in head injury patients at WKS risk. | Alcohol history association with administration. | Clinical signs (e.g., ataxia, confusion), lab tests (LFTs, hematology), glucose loads, thiamine treatment. | No protocols; inconsistent thiamine use; retrospective design; small sample; incomplete thiamine status data. |
| **Guirguis**  2017 | 44,68 | Inpatient | Anxiety disorders, depressive disorders, bipolar disorders, and psychotic disorders | Diabetes, HIV, cancer, dementia, epilepsy, BMI ≥ 40, and chronic renal failure | Retrospective chart review for evidence of alcohol dependence | NA | NR | Caine Criteria | WE frequency and management in psychiatric inpatients. | None explicitly stated. | Caine's diagnostic criteria, retrospective chart review. | Retrospective; single-site; small sample; high underdiagnosis/undertreatment rates; difficulty in post-discharge follow-up. |
| **Isenberg-Grzeda**  2014 | 49 ± 6,5 | Inpatient | 4.2% of the patients had neuro/psychiatric comorbidities | cardiovascular (28.4%), metabolic (18.0%), gastrointestinal (17.5%), infectious (16.9%), hepatic (15.8%), and respiratory (8.7% | Referred to addiction psychiatry service for AUD | NA | NR | Inferred Methods (patients with alcohol use disorders and high-risk individuals based on comorbid conditions like alcohol intoxication, withdrawal, or delirium tremens) | Thiamine prescribing for inpatients with AUD. | None mentioned. | Medical records analysis, Chi-square tests. | Single hospital; no U.S. thiamine dosing guidelines; small sample relative to national scope. |
| **Lin**  2019 | 48 ± 13 | Inpatient | affective disorder (91%) and schizophrenia spectrum disorder (3%) | Charlson comorbidity score 2,3 in Caine positive group | Patients from an inpatient psychiatric unit with history of alcohol use | AUDIT score | Severe | Caine Criteria | Prevalence of Caine-positive WKS among psychiatric inpatients; neurocognitive improvement post-IV thiamine. | Frequency of substance use and related memory/coordination impairments. | MoCA, 5-item delayed recall, gait/coordination tests, oculomotor examination. | Small sample size; single-center study; self-reported substance use; no diagnostic biomarkers; underreporting likely. |
| **Linder**  2018 | 48 ± 11,5 | Inpatient | Major depressive disorder (25%), Anxiety disorder (14%) | - Gastrointestinal disease or procedure - Cancer - Conditions with recurrent vomiting - Malnourished states - Neurocognitive deficits - Systemic disease (eg, HIV/AIDS) | ICD-9 | NA | NR | Caine Criteria | Thiamine use characterization in AUD patients and WE risk evaluation. | None explicitly stated. | Retrospective chart review, χ² analysis, ICD-9 coding. | Small parenteral group; retrospective design; no baseline thiamine levels; psychiatric patients only. |
| **McIntosh**  2005 | *41-50 (median) | Inpatient | NR | NR | Retrospective chart review of inpatients admitted to the Alcohol Problems Clinic for alcohol detoxification | NA | NR | Classic/ICD Criteria | Adherence to thiamine guidelines post-intervention. | Improved outcomes in WKS risk patients. | Case note audits, logistic regression, symptom records. | Retrospective case-note study; likely underestimation of need; population differences; small sample size. |
| **Mifsud**  2022 | *56 (49-64) (median, Q1-Q3) | Inpatient | Mood disorders (12,5%) | 88% of patients had at least one comorbidity, including liver, lung, heart, kidney, pancreatic, neurological, or psychiatric diseases | History of alcohol use documented in medical records, assessed retrospectively during case review | NA | NR | Caine criteria | Clinical and radiological heterogeneity in thiamine deficiency. | Neurological and systemic symptom evaluation. | Brain MRI, clinical criteria, nutritional assessment (BMI, serum albumin). | Single-center retrospective design; variability in thiamine protocols; limited statistical power. |
| **Nakamura**  2018 | 54 ± 14 | Inpatient | NR | NR | Patients at risk of thiamine deficiency; alcohol use not explicitly stated but inferable as a primary cause due to study context | NA | NR | Inferred Methods (altered mental status) | Thiamine prescribing practices and outcomes in encephalopathy patients. | HDIV thiamine association with clinical characteristics and mortality. | Electronic medical records, logistic regression, Chi-square tests. | Retrospective; few receiving guideline-concordant doses; potential unmeasured confounders. |
| **Novo-Veleiro**  2023 | 54.6 years (range 20.9–89.0 years)* median | Inpatient | NR | Malnutrition (30.4%), folic acid deficiency (34.7%), and other deficiencies are highlighted. | High-risk alcohol consumption (>28 SDU/week or 280 g ethanol/week for men and >17 SDU/week or 170 g ethanol/week for women) or active alcohol consumption with a previously established diagnosis. | NA | NR | Caine criteria | Thiamine dosage, frequency, and administration effects on WE recovery/mortality. | Clinical features (e.g., altered mental status, folic acid deficiency) and outcomes. | Caine criteria, MRI, biochemical markers, ICD coding, statistical analysis (χ² test, logistic regression). | Retrospective design; treatment variability; bias toward severe cases; no long-term follow-up. |
| **Ramayya**  1997 | 53,4 ± 8,78 | Inpatient | NR | NR | Patient case records, including alcohol histories, medical histories, psychiatric histories | NA | NR | Classic/ICD Criteria | Korsakoff's psychosis incidence and characteristics in Glasgow (1990-1995). | Male-to-female ratio, response to treatment, alcohol consumption patterns. | ICD-9/ICD-10, DSM-IV criteria, demographic and clinical records (SMR). | Retrospective; small catchment area; underdiagnosis likely; limited nutritional data; anaphylaxis concerns with parenteral thiamine. |
| **Rees**  2013 | 43,3 | Inpatient | NR | mention factors such as diabetic status and liver function test results but does not explicitly report non-psychiatric comorbidities. | DSM-IV | Daily alcohol intake | Severe | Inferred Methods (blood thiamine level tests) | Mandatory thiamine enrichment impact on blood levels in alcohol-dependent individuals. | Dietary intake, supplementation, and blood thiamine levels. | HPLC, dietary interviews, blood tests. | Small control group (n=20); dietary intake not quantified; alcohol effects on thiamine uptake not addressed. |
| **Sgouros**  2004 | 42,36 ± 11,65 | Inpatient | NR | 86.2% presented with clinical and/or laboratory evidence of liver dysfunction | ICD-10 | SADQ + MAST scores | Severe | Inferred Methods (red blood cell thiamine) | Development of the Thiamine Deficiency Questionnaire (TDQ). | TDQ predictive power and impact of nutritional/clinical variables. | TPP concentration (HPLC), MAST, BMI, GGT. | Small sample (n=58); single-center; requires validation in larger populations. |
| **Silva**  2023 | 55 ± 13,3 | Inpatient | NR | Liver steatosis, steatohepatitis, cirrhosis, peripheral neuropathy | Consumption of more than 280-g alcohol/week for men and 170-g alcohol/week for women | NA | NR | Caine criteria | Timing of imaging and thiamine replacement effects on MRI findings in WE. | Correlation between poor diet and specific MRI hyperintensities. | MRI, clinical data, statistical models (Kolmogorov–Smirnov test). | Retrospective design; small sample size; single-center data; variable MRI protocols. |
| **Sinclair**  2023 | *45 (median) | Inpatient | mood disorders (28%), personality disorders (22%), and neurotic disorders (19%) | Alcohol-related liver disease (13%), Epilepsy (1%), Sleep disorders (1%) | ICD-10 | Daily alcohol intake + duration of alcohol use | NR | Inferred Methods (clinical signs and symptoms documented in patient records, such as disorientation/confusion, ataxia, and ophthalmoplegia/nystagmus) | Comparison of MAAW in mental health vs. addiction units. | Evidence-based practices in MAAW (e.g., thiamine use, relapse prevention). | Clinical records audit, SPSS data analysis, bespoke data collection tool. | Small subgroups; reliance on documentation; potential bias; non-generalizable outside UK. |
| **Talbot**  2011 | 42,5 ± 7,56 | Inpatient | NR | Cirrhosis in 1 case | CAGE | CIWAr score | Mild-Moderate | Classic/ICD Criteria | Efficacy of oral thiamine in preventing WE in alcohol withdrawal. | Time-to-treatment impact and therapy compliance. | CIWA-AR score, CAGE assessment, random glucose, urine ketones. | Small sample; single-center jail setting; protocol adherence impacts results. |
| **Tallaksen**  1992 | 50 | Inpatient | NR | chronic pancreatitis (4), alcoholic cirrhosis (3), alcoholic fatty liver(2), esophagitis (I), seizures (I), delirium tremens (2), diabetes (4), polyneuropathy (15), stroke (I), alcoholic dementia (I), congestive heart failure (3), epilepsy (I), chronic obstructive respiratory disease (I), and pneumonia (I). | Patients fulfilled the criteria for alcohol dependence syndrome, but the framework or reference (e.g., DSM or ICD) was not explicitly stated. | NR | NR | Classic/ICD Criteria | Thiamine/phosphate ester concentrations pre-/post-treatment. | Clinical condition associations (e.g., polyneuropathy, WE). | HPLC, biochemical assays, clinical history/examinations. | Small sample; retrospective; baseline thiamine variability; no long-term follow-up. |
| **Wood**  1977 | 49,7 ± 10 | Inpatient | NR | 30.1% had liver disease. | Chronic alcohol intake assessed as greater than 100 grams of ethanol per day. | Daily alcohol intake + alcohol-related comorbities | Severe | Classic/ICD Criteria | Biochemical evidence of thiamine deficiency in alcoholics. | WE incidence (11.3%). | Erythrocyte transketolase assay, thiamine pyrophosphate effect, urinary thiamine excretion. | Limited to chronic alcoholics; single-site study; no age/sex-matched controls; difficulty correlating TPP effect. |

**Supplementary Table C. Risk of Bias—Cohort Studies (Newcastle–Ottawa Scale)**

|  | **Selection** | | | |  | **Comparability** |  | **Outcome** | | |  |  |
| --- | --- | --- | --- | --- | --- | --- | --- | --- | --- | --- | --- | --- |
| Study | Representativeness of the exposed cohort | Selection of the non exposed cohort | Ascertainment of exposure | Demonstration that outcome of interest was not present at start of study |  | Comparability of cohorts on the basis of the design or analysis |  | Assessment of the outcome | Was follow-up long enough for outcomes to occur | Adequacy of follow up of cohorts |  | Total stars |
|  |  |  |  |  |  |  |  |  |  |  |  |  |
|  |  |  |  |  |  |  |  |  |  |  |  |  |
| Ramayya *et al.* 1997 | ★ |  | ★ | ★ |  |  |  | ★ | ★ | ★ |  | 6 |
| Guirguis *et al.* 2017 | ★ | ★ | ★ |  |  | ★ |  | ★ | ★ | ★ |  | 6 |
| Linder *et al.* 2018 | ★ |  | ★ | ★ |  |  |  | ★ | ★ | ★ |  | 6 |
| Nakamura *et al.* 2018 | ★ |  | ★ |  |  | ★ |  | ★ | ★ | ★ |  | 5 |
| Mifsud *et al.* 2022 | ★ |  | ★ |  |  | ★★ |  | ★ | ★ | ★ |  | 6 |
| Novo-Veleiro *et al.* 2023 | ★ |  | ★ | ★ |  | ★★ |  | ★ | ★ |  |  | 7 |
| Silva *et al.* 2023 | ★ | ★ | ★ |  |  | ★★ |  | ★ | ★ | ★ |  | 7 |

Footnote: NOS domains: Selection (max 4★), Comparability (max 2★), Outcome (max 3★); total 0–9★.

**Supplementary Table D. Risk of Bias—Cross-Sectional Studies (Newcastle–Ottawa Scale)**

|  | **Selection** | | | | **Comparability** |  | **Outcome** | |  |  |
| --- | --- | --- | --- | --- | --- | --- | --- | --- | --- | --- |
| Study | Representativeness of the exposed sample | Non-respondents | Ascertainment of the exposure (risk factor) |  | The subjects in different outcome groups are comparable, based on the study design or analysis. Confounding factors are controlled |  | Assessment of the outcome | Statistical test |  | Total stars |
|  |  |  |  |  |  |  |  |  |  |  |
|  |  |  |  |  |  |  |  |  |  |  |
| Wood *et al.* 1977 | ★ |  | ★ |  |  |  | ★ |  |  | 3 |
| Ferguson *et al.* 2000 | ★ |  | ★ |  | ★ |  | ★ |  |  | 4 |
| Sgouros *et al.* 2004 | ★ |  | ★ |  | ★★ |  | ★ | ★ | | 6 |
| Damsgaard *et al.* 2010 | ★ |  | ★ |  | ★★ |  | ★ | ★ | | 6 |
| Blackmore *et al.* 2014 | ★ |  | ★ |  | ★ |  | ★ | ★ | | 5 |
| Isenberg-Grzeda *et al.* 2014 | ★ |  | ★ |  | ★★ |  | ★ | ★ | | 6 |
| Day *et al.* 2015 | ★ |  | ★ |  | ★ |  | ★ | ★ | | 5 |
| Coulbault *et al.* 2019 | ★ |  | ★ |  | ★★ |  | ★ | ★ | | 6 |
| Collet *et al.* 2019 | ★ |  | ★ |  | ★★ |  |  | ★ | | 5 |
| Sinclair *et al.* 2023 | ★ |  | ★ |  | ★★ |  | ★ | ★ | | 6 |

Footnote: NOS domains and star allocation as above; item-level judgments per study.

**Supplementary Figure A. Risk of Bias—Randomized Trials (ROB-2; per-protocol)**

Caption: Traffic-light summary across domains (randomization, deviations, missing data, measurement, selective reporting).

**Supplementary Table E. Risk of Bias—Non-Randomized Studies of Interventions (ROBINS-I)**

| Study | Confounding | Measurement of intervention | Selection | Deviation from intended intervention | Missing data | Measurement of outcomes | Reported results |  | Overall |
| --- | --- | --- | --- | --- | --- | --- | --- | --- | --- |
| Baron *et al.*, 2024 | Moderate | Moderate | Low | Moderate | Low | Low | Low |  | Moderate |
| Baker *et al.*, 1976 | Serious | Low | Moderate | Low | Serious | Serious | Moderate |  | Serious |
| Tallaksen *et al.*, 1992 | Serious | Low | Moderate | Low | Moderate | Serious | Moderate |  | Serious |
| McIntosh *et al.*, 2005 | Serious | Low | Moderate | Moderate | Moderate | Moderate | Moderate |  | Serious |
| Talbot *et al.*, 2011 | Serious | Low | Moderate | Moderate | Moderate | Moderate | Moderate |  | Serious |
| Lin *et al.*, 2019 | Serious | Low | Moderate | Moderate | Moderate | Serious | Moderate |  | Serious |
| Rees *et al.*, 2013 | Serious | Low | Moderate | Moderate | Moderate | Serious | Moderate |  | Serious |

Footnote: Domain judgments: confounding, classification of interventions, selection, deviations from intended interventions, missing data, measurement of outcomes, reporting of results; overall risk shown in last column.
